# *phylogenize*: correcting for phylogeny reveals genes associated with microbial distributions

**DOI:** 10.1093/bioinformatics/btz722

**Published:** 2019-10-07

**Authors:** Patrick H Bradley, Katherine S Pollard

**Affiliations:** 1 Gladstone Institute of Data Science and Biotechnology, San Francisco, CA 94158, USA; 2 Department of Epidemiology and Biostatistics, University of California, San Francisco, CA 94158, USA; 3 Chan–Zuckerberg Biohub, San Francisco, CA 94158, USA

## Abstract

**Summary:**

Phylogenetic comparative methods are powerful but presently under-utilized ways to identify microbial genes underlying differences in community composition. These methods help to identify functionally important genes because they test for associations beyond those expected when related microbes occupy similar environments. We present *phylogenize*, a pipeline with web, QIIME 2 and R interfaces that allows researchers to perform phylogenetic regression on 16S amplicon and shotgun sequencing data and to visualize results. *phylogenize* applies broadly to both host-associated and environmental microbiomes. Using Human Microbiome Project and Earth Microbiome Project data, we show that *phylogenize* draws similar conclusions from 16S versus shotgun sequencing and reveals both known and candidate pathways associated with host colonization.

**Availability and implementation:**

*phylogenize* is available at https://phylogenize.org and https://bitbucket.org/pbradz/phylogenize.

**Supplementary information:**

Supplementary data are available at *Bioinformatics* online.

## 1 Introduction

Shotgun and amplicon sequencing allow previously intractable microbial communities to be characterized and compared, but translating these comparisons into gene-level mechanisms remains difficult. Researchers typically correlate microbial gene abundances with environments using metagenomes, either from shotgun sequencing (Nayfach and Pollard, 2016) or imputed from amplicon sequences (Aßhauer *et al.*, 2015; Langille *et al.*, 2013). However, related microbes tend to both share genes and occupy similar environments, causing spurious correlations. Phylogenetic methods can correct for such confounding in metagenomics data (Bradley *et al.*, 2018), but are currently implemented only in command-line, computationally intensive software.

We developed *phylogenize*, a pipeline allowing researchers without specific expertise in phylogenetic regression to analyze their own data via the web, an R package (R Core Team, 2017), or the popular microbiome workflow tool QIIME 2 (Bolyen *et al.*, 2019). An important innovation specific to *phylogenize* is that input data can be shotgun metagenomes or 16S amplicon data, the latter being lower-cost and available for more environments. Using these taxonomic profiles and sample environments (i.e. sources), the tool returns genes associated with differences in community composition across environments.

## 2 Overview

Users provide *phylogenize* with taxon abundances and sample annotations, in tabular or BIOM (McDonald *et al.*, 2012) format. Shotgun data should be mapped to species using MIDAS (Nayfach *et al.*, 2016). MIDAS defines microbial species based on genome clustering and uses single-copy, universal bacterial gene families (Wu *et al.*, 2013) to estimate taxon abundances; MIDAS also yields strain-specific information for species with sufficient coverage, but only species-level data are required for *phylogenize*. Amplicon data should be denoised to amplicon sequence variants (ASVs) with DADA2 or Deblur. *phylogenize* uses BURST (Al-Ghalith and Knights, 2017) to map ASVs to MIDAS species via individual PATRIC genomes (Wattam *et al.*, 2014), using a default cutoff of 98.5% nucleotide identity (Rodriguez-R *et al.*, 2018) and summing reads mapping to the same species. Taxa are linked to genes using MIDAS and PATRIC, and then gene presence is tested for association with one of two possible phenotypes: prevalence (frequency microbes are observed) or specificity (enrichment of microbes relative to other environments; see Bradley *et al.*, 2018).

*phylogenize* is an R package with a QIIME 2 wrapper written in Python and a web front-end written in Python with the Flask framework (https://www.palletsprojects.com/p/flask) and a Beanstalk-based queueing system (https://beanstalkd.github.io). *phylogenize* reports include interactive trees showing the phenotype’s phylogenetic distribution, heatmaps of significantly positively associated genes, tables showing which SEED subsystems (Overbeek *et al.*, 2005) are significantly enriched, and links to tab-delimited files containing complete results.

## 3 Example applications

### 3.1 Human Microbiome Project

**Fig. 1.**
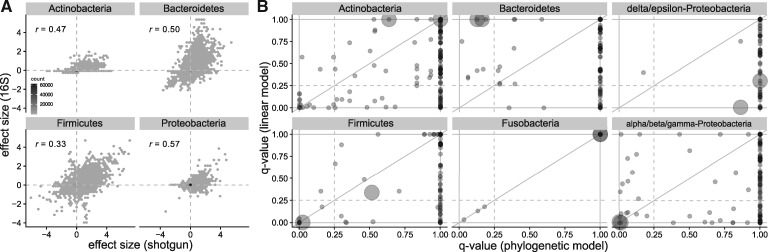
(**A**) Effect sizes computed with *phylogenize* based on HMP shotgun (*x*-axis) and 16S amplicon (*y*-axis) data are correlated. FIGfam gene families with *q *< 0.05 in one or both analyses shown with their Pearson correlation. Examples of SEED subsystems enriched for positively associated genes with both data types include ‘Sporulation gene orphans’ in Firmicutes ($q_{\text{shotgun}}=3.1\times 10^{-12},\;q_{16S}=1.3\times 10^{-18}$) and ‘Type III, Type IV, Type VI, ESAT secretion systems’ in Proteobacteria ($q_{\text{shotgun}}=6.9\times 10^{-38},\;q_{16S}=7.0\times 10^{-8}$). (**B**) SEED enrichments in EMP data using *phylogenize* (*x*-axis; 61 subsystems) or a linear model (*y*-axis; 200 subsystems). Larger circles represent the terms ‘nitrogen fixation’ and ‘nitrogen metabolism’. Full list of enrichments in Supplementary Table S1

The Human Microbiome Project (HMP; Human Microbiome Project Consortium, 2012) collected both 16S amplicon and shotgun sequences from 16 body sites on 192 individuals. Shotgun data processing was previously described (Bradley *et al.*, 2018). Amplicon samples (*n* = 6577) were downloaded from the NCBI SRA and denoised with DADA2 (Callahan *et al.*, 2016), combining reads from the same individual and site. We ran *phylogenize* on both data types to identify genes whose presence is associated with prevalence in the gut. Despite differing read depth and sequencing technology (454 versus Illumina), effect sizes for genes associated with gut prevalence were similar for amplicon and shotgun (0.33≤r≤0.57) and similar pathways were enriched (Fig. 1A).

### 3.2 Earth Microbiome Project

The Earth Microbiome Project (EMP; Thompson *et al.*, 2017) comprises 16S data from many biomes and habitats. Using the balanced subset of 2000 samples processed using Deblur (Amir *et al.*, 2017), we ran *phylogenize* and linear models (no phylogenetic correction) to identify genes whose presence is specific to plant rhizosphere compared to other environments. Linear models identified many more positively associated genes (24 728 versus 7490, q≤0.05) and SEED subsystem enrichments (200 versus 61 subsystems, q≤0.25). However, though nitrogen fixation is a key function of plant rhizospheres (Mylona *et al.*, 1995), fewer linear model enrichments were in the terms ‘nitrogen fixation’ or ‘nitrogen metabolism’ (4/200 versus 5/61 or 2.0% versus 8.2%; Fig. 1B). Since significant genes unique to the linear model were also more correlated with phylogeny in 4 out of the 5 phyla (Ives-Garland α=24.0 versus 11.4, $p\leq 2\times 10^{-16}$: Supplementary Fig. S1), this suggests dilution by false positives.

## 4 Conclusion

Many microbes of interest to clinicians, ecologists and microbiologists are poorly characterized or experimentally intractable. By making it easier to analyze either 16S or shotgun data with more precise statistical tools, *phylogenize* expands the toolkit for identifying mechanisms driving differences in microbial community composition.

## Supplementary Material

btz722_Supplementary_Data
