# Supplementary material for: phylogenize: correcting for phylogeny reveals genes associated with microbial distributions
Source: Bioinformatics. 2019 Oct 7;36(4):1289–90. doi: 10.1093/bioinformatics/btz722 (PMC7703751; doi:10.1093/bioinformatics/btz722)
Supplement: btz722_Supplementary_Data [file btz722_supplementary_data.zip › SuppFig1.pdf]

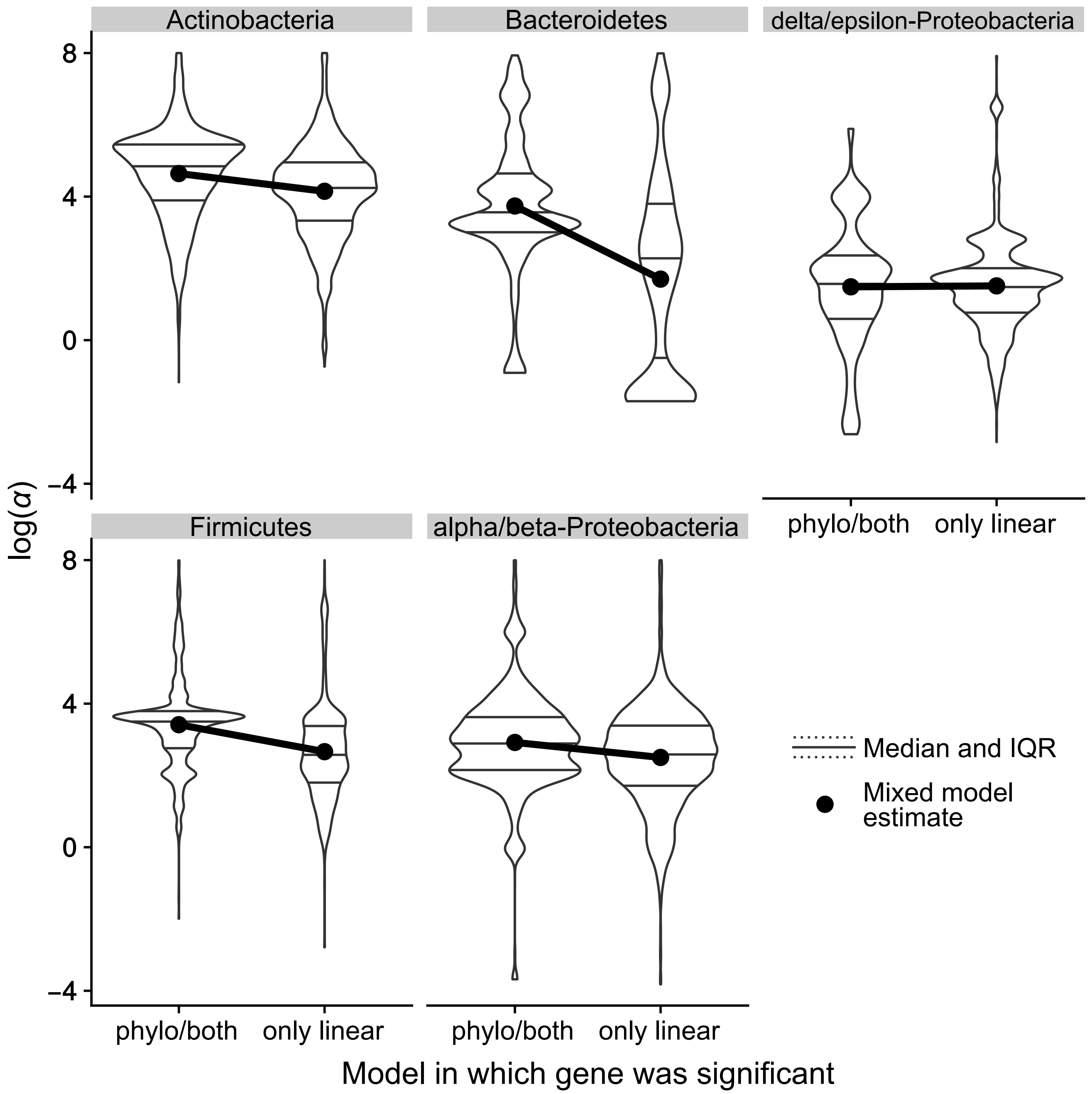

Supplemental Figure 1: Rhizosphere-associated gene families that were only significant in the linear model were enriched for phylogenetic signal in four out of five phyla. Ives-Garland α was estimated for genes that were significantly associated with the plant rhizosphere environment using either a standard linear model or *phylogenize* (violin plots; from top to bottom, lines represent 75th, 50th, and 25th percentiles). Note that α represents the rate of "switching" between gene presence and absence along the tree, so smaller values represent *more* phylogenetic signal. Using linear regression, the distributions of $\log(\alpha)$ gene families that were significant in the phylogenetic model or both models ("phylo/both") were compared to those that were significant in only the linear model ("only linear") with phylum-specific interaction terms (since only four interaction terms could be fitted, we chose to drop the Firmicutes interaction term, which was the closest to zero). All effects were tested for significance with a t-test, with resulting p-values corrected with a Bonferroni-Holm correction. The main effect of "only linear" across all phyla was estimated at -0.74 ± 0.014, indicating that on a linear scale, the "phylo/both" significant genes had $\alpha = 24.0$ (95% CI: 21.7–26.4 and the "only linear" genes had $\alpha = 11.4$ (95% CI: 10.8–12.1). A t-test on the main effect yielded a corrected $p \leq 2 \times 10^{-16}$. All four fitted interaction terms were significant. The main effect was attenuated slightly in Actinobacteria (effect size 0.18, corrected $p = 1.02 \times 10^{-4}$) and alpha/beta/gamma-Proteobacteria (effect size 0.29, corrected $p = 7.93 \times 10^{-12}$), exaggerated in Bacteroidetes (effect size -1.28, corrected $p \leq 2 \times 10^{-16}$, and reversed in delta/epsilon-Proteobacteria (effect size 1.21, corrected $p \leq 2 \times 10^{-22}$).
